# Prevalence and Risk Factors of *Borrelia burgdorferi* Sensu Lato IgG Antibodies Among Blood Donors in Western Romania

**DOI:** 10.3390/pathogens15020125

**Published:** 2026-01-23

**Authors:** Alin Gabriel Mihu, Maria Daniela Mot, Daniela Adriana Oatis, Sergiu Adrian Sprintar, Liana Maria Chicea, Rodica Lighezan, Ana Alexandra Ardelean, Maria Alina Lupu, Tudor Rares Olariu

**Affiliations:** 1 Center for Diagnosis and Study of Parasitic Diseases, Department of Infectious Disease, Victor Babes University of Medicine and Pharmacy, 300041 Timisoara, Romania; alin.mihu@umft.ro (A.G.M.); daniela.oatis@umft.ro (D.A.O.); sergiu.sprintar@umft.ro (S.A.S.); lighezan.rodica@umft.ro (R.L.); rolariu@umft.ro (T.R.O.); 2 Patogen Preventia, 300124 Timisoara, Romania; 3 Aurel Ardelean Institute of Life Sciences, Vasile Goldis Western University of Arad, 310414 Arad, Romania; 4 Department of Biology and Life Science, Faculty of Medicine, Vasile Goldis Western University of Arad, 310025 Arad, Romania; 5 Department of Medicine, Vasile Goldis Western University of Arad, 310414 Arad, Romania; mot.dana@uvvg.ro; 6 Department II Medical Clinic, “Victor Papilian” Faculty of Medicine, Lucian Blaga University of Sibiu, 550024 Sibiu, Romania; liana.chicea@gmail.com; 7 Internal Medicine Department, Academic Emergency Hospital, 550245 Sibiu, Romania; 8 Discipline of Parasitology, Department of Infectious Diseases, Victor Babes University of Medicine and Pharmacy, 300041 Timisoara, Romania; 9 Regional Blood Transfusion Center, 300737 Timisoara, Romania; 10 Clinical Laboratory, Municipal Clinical Emergency Hospital, 300254 Timisoara, Romania; 11 Clinical Laboratory, Institute of Cardiovascular Diseases, 300310 Timisoara, Romania

**Keywords:** *Borrelia burgdorferi*, seroprevalence, blood donors, Western Romania

## Abstract

*Borrelia burgdorferi* sensu lato is a complex of spirochetes that includes the main pathogenic species *B. burgdorferi* sensu stricto, *B. afzelii*, and *B. garinii*, the causative agents of Lyme disease. Our aim was to determine the seroprevalence of anti-*Borrelia* IgG antibodies and assess associated risk factors among blood donors from Western Romania. We conducted a cross-sectional study of 1347 consecutive donors at the Regional Blood Transfusion Center in Timisoara, Western Romania, between November and December 2018. Participants completed an epidemiological questionnaire and serum samples were tested for IgG antibodies against *B. burgdorferi* sensu lato using the VIDAS^®^ Lyme IgG assay. The overall seroprevalence was 2.08% (28/1347). Individuals aged 46–55 years had the highest prevalence (3.79%) and a more than fivefold increased risk compared to those aged 18–25 years (aOR = 4.77; 95% CI: 1.24–18.27; *p* = 0.023). Soil exposure was also independently associated with higher seropositivity (aOR = 2.37; 95% CI: 1.10–5.09; *p* = 0.027). Other factors, including residence, gender, and pet ownership, showed no significant associations. Our findings provide new epidemiological data for Romania and emphasize the importance of environmental exposures in shaping *Borrelia* seroprevalence.

## 1. Introduction

*Borrelia burgdorferi* sensu lato (*B. burgdorferi* s.l.) is a complex of spirochetes that include several species responsible for Lyme disease. The main pathogenic species include *B. burgdorferi* sensu stricto, *B. afzelii*, and *B. garinii* [1]. These spirochetes are tick-borne obligate pathogens that persist in small mammals and birds, which act as natural reservoirs without developing the disease. In humans, however, infection can lead to Lyme borreliosis. Transmission of *B. burgdorferi* most commonly occurs through ticks of the *Ixodes* (*I.*) genus, including *I. scapularis* (the black-legged tick), *I. pacificus*, *I. ricinus*, *I. persulcatus* [2,3,4]. In Europe, the primary vector is *I. ricinus*, whereas in North America transmission is mainly through *I. scapularis* [5].

In humans, *B. burgdorferi* s.l. infection produces diverse clinical manifestations, ranging from early localized erythema migrans to disseminated forms such as Lyme arthritis and carditis, and late neurological complications (Lyme neuroborreliosis), including encephalomyelitis and cognitive impairment [3,6,7,8,9]. The infection begins when spirochetes are transmitted into the dermis with tick saliva during feeding, a process that protects them from immediate exposure to the host’s defense factors [10]. Following deposition, spirochetes disseminate through extracellular matrix components, aided by adhesins such as decorin-binding proteins A and B (DbpA/B) and fibronectin-binding proteins (BBK32). During vascular interactions, they tether and roll along endothelial cells under shear stress, mediated by BBK32 and P66, which facilitate extravasation and dissemination into tissues [5,11,12]. Colonization of immunoprotective niches allows persistence in joints, skin, heart, and nervous system, producing clinical outcomes ranging from erythema migrans to arthritis, carditis, and neuroborreliosis. Persistence is further ensured by multiple immune evasion strategies as complement inhibition by outer surface protein C (OspC), cold shock protein A (CspA), and complement-regulator acquiring surface proteins (CRASPs), suppression of innate responses, and antigenic variation at the variable major protein-like sequence, expressed (*VlsE*) locus. These combined mechanisms enable *B. burgdorferi* to establish chronic infection and present the variable clinical manifestations found in Lyme disease [5,13,14].

The diagnosis of Lyme borreliosis is primarily clinical in the early stage, with erythema migrans considered sufficient for confirmation in endemic regions [15]. For other clinical manifestations, laboratory testing is required, most commonly using the two-tiered approach consisting of an initial enzyme immunoassay (EIA) and enzyme-linked immunosorbent assay (ELISA) followed by a confirmatory immunoblot [16]. While serology has limited sensitivity in early localized infections, immunoglobulin G (IgG) antibody detection becomes reliable in later stages, as these antibodies may persist for months or years after exposure [16,17]. Because of their long-term persistence, anti-*B. burgdorferi* s.l. IgG antibodies are widely used in seroepidemiological surveys to assess past exposure at the population level, even though such studies cannot distinguish between previous and current infection [18,19].

Lyme disease is the most common tick-borne infection in the Northern Hemisphere, with epidemiological studies documenting its presence in North America, Europe, and parts of Asia. In the United States (U.S.), approximately 30,000 cases are reported annually to the Centers for Disease Control and Prevention (CDC), though the true incidence is estimated to be nearly ten times higher [20]. Most U.S. cases occur in the Northeast, Mid-Atlantic, and Upper Midwest, while Canada has also documented increasing numbers, with 917 cases reported in 2015 [20]. In Europe, national surveillance systems report an estimated 85,000 cases of Lyme borreliosis each year, with the highest incidence in central countries such as Germany, Austria, Slovenia, and parts of Sweden [21]. According to a recent review by Davidson et al., [22] approximately 132,000 Lyme borreliosis cases are reported annually in European countries with surveillance systems. The study also showed that incidence is highest in countries reporting both clinical and laboratory-confirmed cases, and that nearly 30% of Europeans live in subnational high-incidence areas [22]. In the United Kingdom, approximately 3000 cases are reported annually in England and Wales, while incidence is particularly elevated in the Scottish Highlands [21]. In Asia, human cases have been confirmed across mainland China, while incidence remains low in Japan and Korea [20,23].

Limited data are available to the international medical community regarding the seroepidemiology of *B. burgdorferi* s.l. infection in Romania. Kalmar et al. (2021) tested blood donors from six counties from northwestern and central Romania during 2019–2020 [24]. The seroprevalence was assessed using ELISA and confirmed by Western blot analysis. The rate of specific IgG antibodies was 2.3% and the rate of IgM was 1.8%, with county variation between 0.5 and 3.0% [24]. Hristea et al. (2001) [25] tested blood donors and forestry workers from 13 counties for anti-*B. burgdorferi* s.l. IgG antibodies in 1999. The seroprevalence was determined using passive hemagglutination assays and Western blot analysis. The rate was of 4.3% in blood donors (ranging between 1.4 and 8.7%) and 9.3% in forestry workers [25]. A recent burden analysis reconfirmed that no representative household surveys exist, and blood donor studies remain the main proxy for the general population [26].

No seroepidemiological data on *B. burgdorferi* s.l. are currently available for Timis county, Western Romania. Therefore, the objective of this study was to determine the seroprevalence of anti-*B. burgdorferi* s.l. IgG antibodies and to evaluate potential demographic and environmental risk factors in healthy blood donors from this region.

## 2. Materials and Methods

We conducted a cross-sectional study of 1347 consecutive blood donors at the Regional Blood Transfusion Center in Timisoara, Timis County, Western Romania, between 19 November and 21 December 2018. The western region of Romania comprises four counties: Timiș, Arad, Caraș-Severin, and Hunedoara. Of the 1347 (100%) participants included in the study, 1169 (86.8%) were recruited from Timiș County, with the remaining donors originating from Arad, Caraș-Severin, and Hunedoara counties.

The study included consecutive blood donors who attended the Regional Blood Transfusion Center during the study period, who were willing to participate and to complete a self-administered questionnaire. Exclusion criteria were lack of consent and failure to meet the national blood donation eligibility criteria in Romania, which prohibit donation by individuals with type I diabetes, schizophrenia, epilepsy, chronic hepatitis, liver cirrhosis, human immunodeficiency virus (HIV) infection, cancer, anemia or asthma [27].

**Figure 1:**
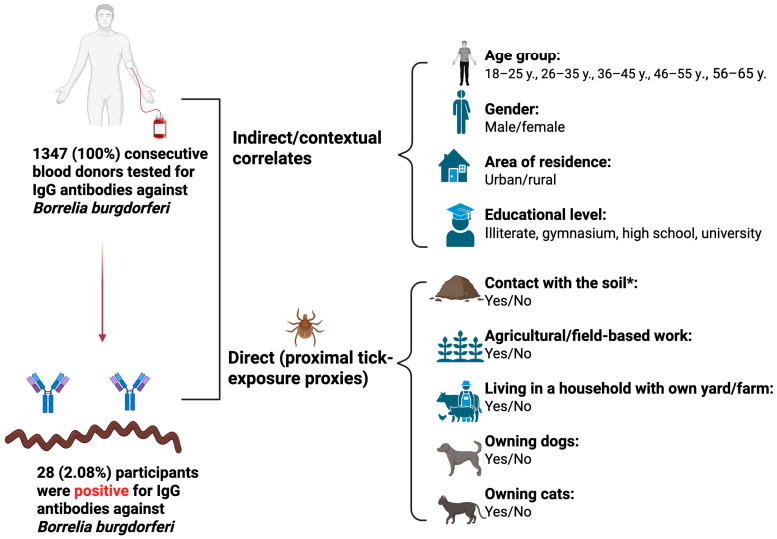
Distribution of IgG seropositivity for *Borrelia burgdorferi* and classification of investigated risk factors into indirect contextual correlates and direct proximal tick-exposure proxies in 1347 blood donors from Western Romania (Created in BioRender. Hermenean, A. (2026) https://BioRender.com/b92ve7e accessed on 15 January 2026). * = The variable “Contact with the soil” refers to activities such as gardening, yard maintenance, and other comparable peridomestic outdoor activities.

For each participant the following demographic characteristics were collected to ensure full anonymity: age group (18–25 years, 26–35 years, 36–45 years, 46–55 years and 56–65 years), gender (male/female), area of residence (urban/rural) (Figure 1). Urban or rural residence was assigned according to the official administrative classification of the participant’s permanent residence.

Participants also completed a self-administered questionnaire under the strict supervision of specialized nurses who had been instructed by the primary investigators. The questionnaire assessed educational level (illiterate, gymnasium, high school, university), current engagement in activities involving soil contact such as gardening, yard work, or other leisure/domestic activities (yes/no), engagement in agricultural or field-based work such as farming, forestry, vineyard work, crop harvesting, or animal husbandry (yes/no), living in a household with own yard/farm (yes/no), and pet ownership (cats or dogs; yes/no) (Figure 1). We used the variable “Contact with the soil” as an umbrella proxy encompassing gardening, yard maintenance, and other comparable peridomestic outdoor activities.

For the purpose of clearly distinguishing their role in Lyme seropositivity, we classified the investigated risk factors into indirect contextual correlates (age group, gender, area of residence, and educational level) and direct proximal tick-exposure proxies (soil contact, agricultural or field-based work, owning cattle, and pet ownership of cats or dogs) (Figure 1).

Venous blood samples were collected using a standard venipuncture technique into serum separator gel and clot activator tubes (Becton Dickinson, Vaud, Switzerland). Within 10–30 min, the samples were centrifuged at 4000× *g* for 10 min, and the resulting serum was aliquoted into sterile Eppendorf tubes and stored at −20 °C until testing.

Sera were analyzed for IgG antibodies against *B. burgdorferi* s.l. at the Center for Diagnosis and Study of Parasitic Diseases, Victor Babes University of Medicine and Pharmacy, Timisoara, Romania.

IgG antibodies against *B. burgdorferi* were determined using the VIDAS^®^ Lyme IgG assay on the VIDAS^®^ automated platform (bioMérieux, Marcy-l’Étoile, France). The principle of the assay combines a 2-step enzyme immunoassay sandwich method with a final fluorescent detection. According to the manufacturer’s instructions, test values < 0.20 were considered negative and values ≥ 0.2 were considered positive. The assay employs recombinant chimeric proteins (*VlsE*, DbpA, and OspC), including the immunodominant C6 peptide region, to detect IgG responses against the main pathogenic *Borrelia* strains. *VlsE* is produced inside the living host, and presents a highly immunogenic region, making it important for serological diagnosis. DbpA is a protein produced on the surface of bacteria while in a living host. OspC is mainly synthesized during the infection in the living host. Reported performance characteristics indicate a sensitivity of approximately 83–85% and a specificity of 85–88% when used as a first-tier test in two-tier Lyme disease diagnosis [28,29].

Statistical analyses were conducted using Stata version 16.1 (StataCorp, College Station, TX, USA). Data are presented as numbers, percentages, and mean ± standard deviation (SD). Associations between seropositivity and potential risk factors were first assessed through univariate logistic regression. Variables with a significance level of *p* < 0.05 were then included in a multivariate logistic regression model to identify independent predictors. Results are reported as crude odds ratios (cOR) for univariate analyses and adjusted odds ratios (aOR) for multivariate analyses, each with corresponding 95% confidence intervals (CI). A two-tailed *p* value of <0.05 was considered statistically significant.

The study was approved by the Ethics Committee of the *Victor Babes* University of Medicine and Pharmacy, Timisoara (approval No. 4/8 February 2018). Written informed consent for participation was obtained from all participants, and the results of their serological tests were communicated to each individual.

## 3. Results

The 1347 blood donors included in the study were aged between 18 and 63 years (mean age = 33.6; SD = 10.9 years), with 979 (72.7%) residing in urban areas and 755 (56.1%) being males. The overall seroprevalence of *Borrelia burgdorferi* IgG antibodies was 2.08% (28/1347). When compared with participants aged 18–25 years, those aged 46–55 years showed the highest seropositivity (3.79%) and had more than a fivefold increased odds of infection (cOR = 5.36; 95% CI: 1.41–20.42; *p* = 0.014). Seropositivity was slightly higher among rural residents (2.99%; 11/368) compared to urban residents (1.74%; 17/979), but this difference was not statistically significant (OR = 0.57; 95% CI: 0.27–1.24; *p* = 0.156). Male participants showed a higher seroprevalence (2.65%; 20/755) compared to females (1.35%; 8/592), although this association also did not reach statistical significance (OR = 1.99; 95% CI: 0.87–4.54; *p* = 0.104) (Table 1).

Both indirect factors (such as sociodemographic characteristics) and direct exposures related to tick contact were analyzed in relation to *Borrelia burgdorferi* IgG seropositivity. Individuals aged 46–55 years showed the highest prevalence (3.79%; 8/211), with odds of infection over five times greater than in the youngest group aged 18–25 years (0.73%; 3/411) (cOR = 5.36; 95% CI: 1.41–20.42; *p* = 0.014). Although increased prevalence was also seen among those aged 26–35 years (2.46%; 10/407) and 36–45 years (2.48%; 7/282), the differences compared to the reference group were not statistically significant (Table 1).

Soil exposure emerged as an important predictor, with seropositivity more than twice as common among exposed individuals (3.52%; 14/398) compared to those without soil contact (1.48%; 14/949) (cOR = 2.43; 95% CI: 1.15–5.16; *p* = 0.020). Likewise, living in a household with its own yard or farm was linked to increased risk (3.54%; 14/395 vs. 1.47%; 14/952), corresponding to an odds ratio of 2.46 (95% CI: 1.16–5.21; *p* = 0.019) (Table 1).

Dog ownership (3.36%; 11/327; cOR = 2.05; 95% CI: 0.95–4.43; *p* = 0.067) and cat ownership (3.72%; 8/215; cOR = 1.81; 95% CI: 0.84–3.91; *p* = 0.129) were also associated with higher seroprevalence, but these results did not reach statistical significance (Table 1).

All variables that were statistically significant in the univariate analysis were subsequently included in a multivariate logistic regression model (Table 2).

After adjusting for potential confounders in the multivariate logistic regression model, only age and soil contact showed independent associations with *B. burgdorferi* seropositivity (Table 2). Participants aged 46–55 years had almost a fivefold increased risk compared to those aged 18–25 years (aOR = 4.77; 95% CI: 1.24–18.27; *p* = 0.023). Elevated odds were also observed in the 26–35 years (aOR = 3.65; 95% CI: 0.99–13.39; *p* = 0.051) and 36–45 years (aOR = 3.40; 95% CI: 0.87–13.26; *p* = 0.079) groups, but these findings did not reach statistical significance. Soil exposure remained a significant predictor, with more than twice the odds of seropositivity compared to individuals without such contact (aOR = 2.37; 95% CI: 1.10–5.09; *p* = 0.027). Due to collinearity, the risk factor living in a household with its own yard or farm was not retained in the model; however, in univariate analysis it showed a comparable association, suggesting that both exposures reflect the same underlying risk (Table 2).

## 4. Discussion

Lyme disease, a tick borne multi-system illness, is mostly spread in temperate countries. However, due to increasing tourism, outdoor activities, reforestation and intrusion of man in the vector’s habitat, the incidence of the disease has increased globally. Higher risk of infection is seen in forestry workers, hikers, travelers in endemic [30].

In the present study, IgG antibodies specific to *B. burgdorferi* s.l., were detected in 2.08% of the blood donors tested. This value is higher than the rate of 0.49% reported between 2021 and 2022 in blood donors from England [31], and lower than the ones reported in other blood donor European studies (4.2% in Scotland [32], 6.2% in Danmark, 2.7% in Hamburg and Würzburg, Germany and 3.4% in Ireland) [33,34,35,36]. Comparing our results with the two previous studies from Romania, we noted similar results with Kalmar et al. (2.3% rate during 2019–2020 in 6 north-western and central counties from Romania) [24], but lower rate than the one reported by Hristea et al. (4.3% in 1999 in 13 counties from Romania) [25]. Consistent with these findings, a recent national burden analysis noted that Romania still lacks true household, population-representative serosurveys and that donor data have served as the main proxy for general exposure [26]. Although our results match those obtained by Kalmar et al. in Romania on blood donors [24], regional variation is a common finding in Europe; for example, in Norway seroprevalence ranged from 0.48% in northern counties to 9.25% in the endemic Vestfold region [37]. In the Alpine region, donor seroprevalence varied considerably, with 7.2% in North Tyrol compared to 1.5% in South Tyrol [38].

In the present study, subjects aged 46–55 years showed higher seropositivity (3.79%) and >5-fold higher odds of IgG anti-*B. burgdorferi* s.l. antibodies when compared with participants aged 18–25 years (*p* = 0.014). Hansen et al. (2024) noted an increasing seroprevalence with age in Danish blood donors [34]. This age-related pattern aligns with European seroepidemiology showing that IgG anti-*B. burgdorferi* antibodies rise across adulthood, consistent with cumulative lifetime exposure to tick bites. In a large German population cohort, each additional year of age increased the odds of IgG seropositivity by ~3%, and the authors explicitly argue that accumulation of exposures likely explains the gradient [39]. In a population- based cross sectional survey performed in the Netherlands, the IgG prevalence increased from 2.6% in subjects between 0 and 19 years to 7.7% in people aged 60–88 years [40]. In a cross-sectional survey among German adults, increasing seropositivity with age was note, with prevalence reaching ~20% in 70–79-year-olds [41]. A global meta-analysis also identified age ≥ 50 years as independently associated with higher odds of *Borrelia* seropositivity [42]. Together, these findings support our age effect and suggest that mid-to-late working-age adults accumulate sufficient exposures to manifest higher background IgG, even when recent infection is uncommon.

In the present study, the area of residence was not significantly associated with *B. burgdorferi* s.l. IgG seropositivity. This result was also noted in Scottish blood donors (5.5% vs. 3.7%) [24,32]. Likewise, Belgian sampling found nearly identical rates in rural and urban donors (2.9% vs. 2.6%), suggesting small effect sizes [43], and broader European reviews emphasize heterogeneity across settings that can obscure modest demographic effects [21]. Although no significant association was noted by Kalmar et al. [24], who noted a tendency of higher rate in blood donors from urban regions. An explanation for that outcome was thought to be the limited access of individuals from rural areas to blood donor centers [24]. It is known that high prevalences of Lyme disease are usually found in rural areas. However, in Romania, foci of *I. ricinus* ticks and small mammals infected with *Borrelia* spp. were noted in urban parks and recreational areas [24,44,45].

In our cohort, gender was not significantly associated with *B. burgdorferi* s.l. IgG seropositivity. Same outcome was also noted by Munro et al. in Scottish blood donors [32]. However, in Danish blood donors, females (3.9%) had a significantly lower rate of antibodies against *B. burgdorferi* than males (8.2%) [34]. Kalmar et al. (2021) [24] noted a tendency of higher *B. burgdorferi* seroprevalence in men, but the difference was not statistically significant. This tendency might be associated with higher exposure to tick bites due to outdoor employment and recreational activities [24].

Soil exposure emerged as an important predictor in our cohort, seropositivity was more than twice as common among those reporting soil contact, and a similar increase was observed for participants living in a household with its own yard or farm. Both variables likely capture peridomestic exposure (gardening, yard work, landscaping) and greater proximity to tick habitat and reservoir hosts around the home. Consistent with this interpretation, a U.S. community study found that yardwork/gardening was among the highest-risk activities for tick bites [46]. Peridomestic intervention trials in the U.S. likewise identified the residential yard as a key exposure setting for Lyme risk, with household-level measures (e.g., acaricide application, fencing) aimed at reducing infections acquired in and around the home [47]. Environmental surveys repeatedly document substantial tick abundance in domestic gardens, especially in rural and suburban settings, providing a direct ecological mechanism for increased human exposure near residences [48]. Broader European seroepidemiological and risk-factor studies also align with higher odds of *Borrelia* exposure among persons who report frequent outdoor activities and more tick bites, reinforcing the plausibility that soil contact and living in a yard/farm setting are proxies for cumulative peri-residential tick encounters [39,40].

Dog and cat ownership in our cohort tended to have higher rates of *B. burgdorferi* s.l. IgG antibodies. This pattern aligns with population evidence showing that pet ownership is not an independent predictor of *Borrelia* seropositivity after adjustment [49] and that IgG prevalence can even be lower among pet owners in blood donors [50]. In community data from U.S., outdoor pet ownership was likewise not associated with a higher odd of attached tick bites [46]. Although pets can increase household tick encounters [51], these exposures do not consistently translate into higher seroprevalence, consistent with our non-significant findings [46,49,50,51].

The value of this study resides not only in the data provided on the seroprevalence and risk factors associated with *B. burgdorferi* infection, but also in highlighting a possible public health risk, given that individuals who were tested positive were active blood donors. Although IgG seropositivity indicates prior exposure to *B. burgdorferi*, the existence of seropositive people among active blood donors might raise questions regarding possible transfusion-related implications. In a previous study it has been demonstrated that mice can contract *B. burgdorferi* by transfusion [52]. The transmission, however, could have been impacted by the inoculation method, which involved direct blood transfusion without the usual blood processing or storage [53,54]. According to certain hypotheses, there might not be enough viable spirochaetes in whole blood to survive in blood storage conditions, or the viability of *B. burgdorferi* may be quickly lost during storage with host-adapted *B. burgdorferi*. This suggests that the risk of transmission through transfusion would only exist in situations where unmodified blood products are used, such as in military contexts [17,32]. Further investigations should focus on evaluating the transmission possibility.

This study has certain limitations. Blood donors represent a selected group of generally healthy individuals within the 18–65 year age range, which may not fully reflect the seroepidemiology of the general population [55]. The distribution of individuals within age categories was uneven with the age group 56–65 years being underrepresented. The absence of seropositive samples in this age range might be attributed to the limited sample size rather than actual lack of exposure. Cross-sectional studies are valuable for identifying associations, but they cannot determine causal relationships or the temporal sequence of events [56]. Additionally, our sample included more males, urban residents, and individuals with higher educational levels, a pattern also observed among Danish blood donors [57], which may limit generalizability. The absence of information on the type of environment in which pets lived (indoor vs. outdoor) should be considered a limitation and addressed in further studies. We did not apply a two-step serological approach (screening assay followed by Western blot confirmation), as used in other studies [31,38], but relied solely on IgG antibodies against *B. burgdorferi*. Although confirmatory two-tier testing is recommended for clinical diagnosis, single-tier assays are widely used in seroepidemiological studies and provide useful estimates at the population level when interpreted with caution [58]. Lastly, the study focused on assessing *Borrelia* seroprevalence at the regional level rather than on individual counties, as the blood donation was more accessible to individuals from Timiș County due to the placement of the regional blood transfusion center. Statistical analysis of data stratified by county could not be performed, as it would not provide meaningful statistical results.

## 5. Conclusions

The present study provides new and valuable insights into the seroprevalence of *B. burgdorferi* s.l. and highlights several risk factors associated with the presence of the antibodies in the blood donors from Timis county, Western Romania. Contact with soil and living in a household with farm/own garden increased the chances of anti-*B. burgdorferi* s.l. antibodies. Future studies should also collect more data on the individuals’ behaviors, tick bite history, tick removal practices to better understand the epidemiology of *Borrelia* spp. and practices associated with the infection. Moreover, studies on the general population should be conducted to estimate the true burden of the infection. Further investigations, with large number of samples from general population, would help provide more precise information on the epidemiology of Borrelia infection and its variations across counties from Western Romania.

## Figures and Tables

**Table 1:** Seroprevalence of *Borrelia burgdorferi* IgG antibodies among 1347 blood donors and associated risk factors from Western Romania (univariate logistic regression analysis).

IgG Antibodies Against *Borrelia burgorferi*				Univariate Logistic Regression		
Indirect/Contextual Correlates	No. of Tested	Positive (%)	Negative (%)	cOR	95% CI	*p* Value
Age Groups:						
18–25	411	3 (0.73)	408 (99.27)		Ref.	
26–35	407	10 (2.46)	397 (97.54)	3.43	0.94–12.54	0.063
36–45	282	7 (2.48)	275 (97.52)	3.46	0.89–13.50	0.074
46–55	211	8 (3.79)	203 (96.21)	5.36	1.41–20.42	0.014
56–65	36	0 (0)	36 (100)		-	
Gender:						
Female	592	8 (1.35)	584 (98.65)		Ref.	
Male	755	20 (2.65)	735 (97.35)	1.99	0.87–4.54	0.104
Area of residence:						
Rural	368	11 (2.99)	357 (97.01)		Ref.	
Urban	979	17 (1.74)	962 (98.26)	0.57	0.27–1.24	0.156
Education level:						
Illiterate	30	0 (0)	30 (100)		-	
Gymnasium	174	6 (4.08)	168 (95.92)	2.24	0.80–6.26	0.123
Highschool	505	12 (2.38)	493 (97.62)	1.53	0.66–3.57	0.326
University	638	10 (1.57)	628 (98.43)		Ref.	
Direct (proximal tick-exposure proxies)						
Contact with the soil *:						
No	949	14 (1.48)	935 (98.52)		Ref.	
Yes	398	14 (3.52)	384 (96.48)	2.43	1.50–5.16	0.02
Agricultural/field-based work:						
No	1343	28 (2.08)	1315 (97.92)		Ref.	
Yes	4	0 (0)	4 (100)		N/A.	
Living in a household with own yard/farm:						
No	952	14 (1.47)	938 (98.53)		Ref.	
Yes	395	14 (3.54)	381 (96.46)	2.46	1.16–5.21	0.019
Owning dogs:						
No	1020	17 (1.67)	1003 (98.33)		Ref.	
Yes	327	11 (3.36)	316 (96.64)	2.05	0.95–4.43	0.067
Owning cats:						
No	1132	20 (1.77)	1112 (98.23)		Ref.	
Yes	215	8 (3.72)	207 (96.28)	1.81	0.84–3.91	0.129
Total	1347	28 (2.08)	1319 (97.92)			≈

Ref. = reference; N/A = not applicable; * = The variable “Contact with the soil” refers to activities such as gardening, yard maintenance, and other comparable peridomestic outdoor activities.

**Table 2:** Multivariate logistic regression model of independent risk factors for *Borrelia burgdorferi* IgG seropositivity in 1347 blood donors from Western Romania.

Variable	Multivariate Logistic Regression		
Age Groups (Ref.: 18–25 Years)	aOR	95% CI	*p* Value
26–35	3.65	0.99–13.39	0.051
36–45	3.4	0.87–13.26	0.079
46–55	4.77	1.24–18.27	0.023
56–65		-	
Contact with soil (Ref.: No) *	2.37	1.10–5.09	0.027

Ref. = reference; * = variables with substantial overlap (contact with soil and Living in a household with own yard/farm); only one retained in the model to avoid collinearity.

## Data Availability

All relevant data are contained within the article.

## Abbreviations

The following abbreviations are used in this manuscript:

*B. burgdorferi* s.l.	*Borrelia burgdorferi* sensu lato
CDC	Centers for Disease Control and Prevention
CRASPs	Complement-regulator acquiring surface proteins
CspA	Cold shock protein A
DbpA/B	Decorin-binding proteins A and B
EIA	Enzyme immunoassay
ELISA	Enzyme-linked immunosorbent assay
HIV	Human immunodeficiency virus
IgG	Immunoglobulin G
U.S.	United States
VlsE	Variable major protein-like sequence, expressed
